# Supplementary material for: LEAP2 reduces postprandial glucose excursions and ad libitum food intake in healthy men
Source: Cell Rep Med. 2022 Mar 30;3(4):100582. doi: 10.1016/j.xcrm.2022.100582 (PMC9043997; doi:10.1016/j.xcrm.2022.100582)
Supplement: Document S1. Figure S1 [file mmc1.pdf]

Cell Reports Medicine, Volume *3*

Supplemental information

LEAP2 reduces postprandial glucose excursions and *ad libitum* food intake in healthy men

Christoffer A. Hagemann, Malene S. Jensen, Stephanie Holm, Lærke S. Gasbjerg, Sarah Byberg, Kirsa Skov-Jeppesen, Bolette Hartmann, Jens J. Holst, Flemming Dela, Tina Vilsbøll, Mikkel B. Christensen, Birgitte Holst, and Filip K. Knop

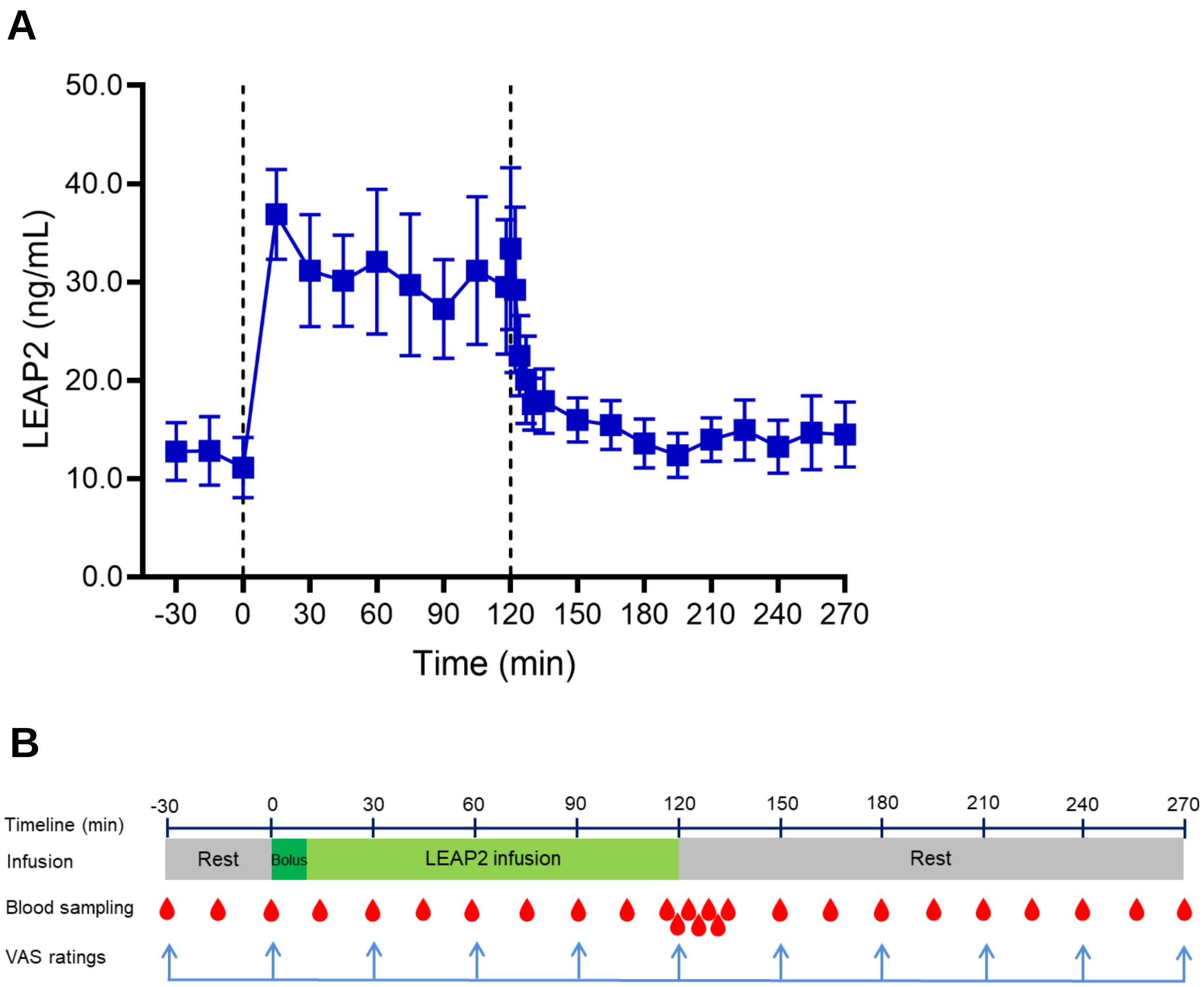

Figure S1. Clinical study describing pharmacokinetic parameters of exogenous LEAP2, Related to STAR Methods.

Plasma concentrations of LEAP2 (**A**) and overview of the clinical study design including eight healthy, young men (**B**). Bold dotted line, infusion start (0 min) and infusion stop (120 min). Data are presented as mean ± SEM. Abbreviations: LEAP2, liver-expressed antimicrobial peptide 2; VAS, visual analogue scale.
