# Supplementary material for: LEAP2 reduces postprandial glucose excursions and ad libitum food intake in healthy men
Source: Cell Rep Med. 2022 Mar 30;3(4):100582. doi: 10.1016/j.xcrm.2022.100582 (PMC9043997; doi:10.1016/j.xcrm.2022.100582)
Supplement: Document S2. Article plus supplemental information [file mmc2.pdf]

Cell Reports Medicine

Article

LEAP2 reduces postprandial glucose excursions and *ad libitum* food intake in healthy men

Graphical abstract

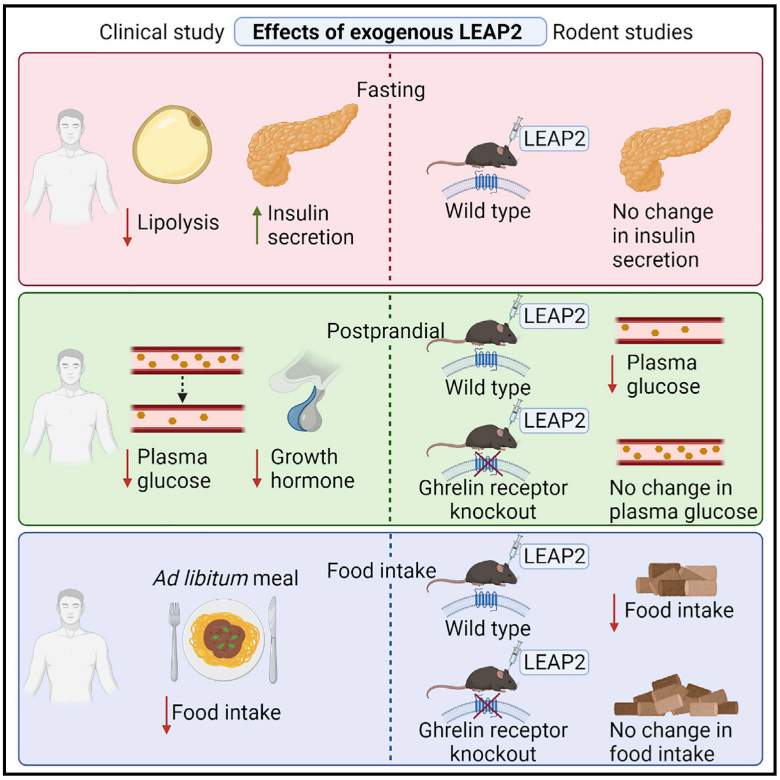

Highlights

- Exogenous LEAP2 lowers postprandial plasma glucose excursions
- Exogenous LEAP2 suppresses *ad libitum* food intake
- During fasting, exogenous LEAP2 increases insulin secretion and suppresses lipolysis
- The GHSR is required for eliciting LEAP2 effects in mice

Authors

Christoffer A. Hagemann, Malene S. Jensen, Stephanie Holm, ..., Mikkel B. Christensen, Birgitte Holst, Filip K. Knop

Correspondence

holst@sund.ku.dk (B.H.), filip.krag.knop.01@regionh.dk (F.K.K.)

In brief

LEAP2 was recently discovered as a hormone in rodents, which modulates ghrelin actions on plasma glucose and appetite. In a randomized, double-blind, placebo-controlled trial, Hagemann et al. demonstrate that exogenous LEAP2 reduces postprandial glucose excursions and food intake in healthy men.

Hagemann et al., 2022, Cell Reports Medicine *3*, 100582
April 19, 2022 © 2022 The Author(s).
https://doi.org/10.1016/j.xcrm.2022.100582

CellPress

Cell Reports Medicine

CellPress
OPEN ACCESS

Article

LEAP2 reduces postprandial glucose excursions and *ad libitum* food intake in healthy men

Christoffer A. Hagemann,[1,2] Malene S. Jensen,[1] Stephanie Holm,[3] Lærke S. Gasbjerg,[1,3] Sarah Byberg,[3] Kirsa Skov-Jeppesen,[3,4] Bolette Hartmann,[3,4] Jens J. Holst,[3,4] Flemming Dela,[3] Tina Vilsbøll,[1,5,6] Mikkel B. Christensen,[1,5,7] Birgitte Holst,[3,*] and Filip K. Knop[1,4,5,6,8,*]
[1]Center for Clinical Metabolic Research, Copenhagen University Hospital Herlev and Gentofte, Hellerup, Denmark
[2]Gubra, Hørsholm, Denmark
[3]Department of Biomedical Sciences, Faculty of Health and Medical Sciences, University of Copenhagen, Copenhagen, Denmark
[4]Novo Nordisk Foundation Center for Basic Metabolic Research, Faculty of Health and Medical Sciences, University of Copenhagen, Copenhagen, Denmark
[5]Department of Clinical Medicine, Faculty of Health and Medical Sciences, University of Copenhagen, Copenhagen, Denmark
[6]Steno Diabetes Center Copenhagen, Gentofte, Denmark
[7]Department of Clinical Pharmacology, Copenhagen University Hospital Bispebjerg and Frederiksberg, Copenhagen, Denmark
[8]Lead contact
*Correspondence: holst@sund.ku.dk (B.H.), filip.krag.knop.01@regionh.dk (F.K.K.)
https://doi.org/10.1016/j.xcrm.2022.100582

SUMMARY

The gastric hormone ghrelin stimulates food intake and increases plasma glucose through activation of the growth hormone secretagogue receptor (GHSR). Liver-expressed antimicrobial peptide 2 (LEAP2) has been proposed to inhibit actions of ghrelin through inverse effects on GHSR activity. Here, we investigate the effects of exogenous LEAP2 on postprandial glucose metabolism and *ad libitum* food intake in a randomized, double-blind, placebo-controlled, crossover trial of 20 healthy men. We report that LEAP2 infusion lowers postprandial plasma glucose and growth hormone concentrations and decreases food intake during an *ad libitum* meal test. In wild-type mice, plasma glucose and food intake are reduced by LEAP2 dosing, but not in GHSR-null mice, pointing to GHSR as a potential mediator of LEAP2's glucoregulatory and appetite-suppressing effects in mice.

INTRODUCTION

The growth hormone (GH) secretagogue receptor (GHSR) modulates fundamental physiological functions, including regulation of food intake, glucose homeostasis, and GH release from the anterior pituitary gland.[1] The gastric hormone ghrelin, an endogenous GHSR agonist, stimulates food intake and gastrointestinal motility and increases plasma glucose.[1,2] Ghrelin, a peptide hormone, requires an acylation to obtain full activity, and its expression is regulated according to energy status.[1,2] Thus, plasma ghrelin concentrations rise during conditions with energy deficit, such as fasting and calorie restriction, and fall after food intake or with obesity.[3,4] Recently, liver-expressed antimicrobial peptide 2 (LEAP2), a 40-amino-acid peptide expressed in the liver and the small intestine, was identified as another GHSR ligand.[5] LEAP2 is both an inverse agonist of GHSR that downregulates the constitutive activity of GHSR and a competitive antagonist that impairs ghrelin-induced activation of GHSR.[4,6,7] Thus, the activity of GHSR is controlled at least in part by two circulating, gut-derived ligands with opposing actions. This type of dual regulation of receptor signaling properties is unusual in human physiology, but it has previously been described for the melanocortin receptors. For example, the melanocortin-4 receptor—an important receptor for appetite regulation—also displays constitutive activity, which is further activated by α-melanocyte-stimulating hormone and inhibited by agouti-related peptide.[8]

LEAP2 is synthesized as a 77-amino-acid prepropeptide and is processed into several truncated forms, including the mature 40-amino-acid residue peptide ($LEAP2_{38–77}$; here called LEAP2), which is identical in mice and humans.[5] Plasma LEAP2 concentrations are regulated inversely compared with plasma ghrelin in several metabolic settings.[4] Hence, LEAP2 concentrations have been reported to decrease during weight loss and increase with obesity.[4] Whether plasma concentrations of LEAP2 increase in response to food intake in humans as seen in rodents remains to be clarified.[4,7] Mani et al.[4] observed a postprandial rise in LEAP2 plasma concentration in obese individuals eligible for bariatric surgery, but not in lean individuals; however, we could not confirm a postprandial increase in LEAP2 plasma levels in a similar group of obese individuals.[7]

Recently, we reported an increased expression level of LEAP2 following the bariatric surgical procedure Roux-en-Y gastric bypass (RYGB) and robust insulinotropic properties *in vitro* of another endogenous LEAP2 fragment, $LEAP2_{38–47}$, however without glucoregulatory effect in a clinical proof-of-concept trial.[7] Interestingly, $LEAP2_{38–47}$ retains the inverse agonistic

© 2022 The Author(s).
This is an open access article under the CC BY license (http://creativecommons.org/licenses/by/4.0/).

Table 1. Baseline characteristics of study participants

Male/female (n/n)	20/0
Age (years)	23 (20–25)
Weight (kg)	80.3 (74.9–88.2)
Height (m)	1.87 (1.81–1.92)
BMI (kg/m^2)	23.1 (22.3–25.0)
Fasting plasma glucose (mmol/L)	5.2 (5.0–5.3)
HbA_{1c} (mmol/mol)	32 (29–33)

Data are presented as median (interquartile range [IQR]). BMI, body mass index; HbA_{1c}, glycated hemoglobin A_{1c}.

properties on GHSR, suggesting that the insulinotropic characteristics of the fragment may be directly linked to downregulation of constitutive GHSR activity.[6,7] Thus, the pharmacological potential of LEAP2 family peptides deserves to be further investigated.

To date, bariatric surgery is the most effective treatment of obesity.[9] Despite documented beneficial effects of individual gut hormones, such as glucagon-like peptide 1 (GLP-1) and peptide YY, on glycemic control and appetite regulation following RYGB surgery,[10,11] the exact mechanisms that regulate changes in gut-derived signals and link them with metabolic control are not well understood. GHSR and ghrelin regulate a wealth of metabolic functions connecting gut and brain and have been considered as possible neuroendocrine therapeutic targets. However, despite two decades of intensive research in the field, no viable clinical candidate has been developed. Consequently, the discovery of the endogenous inverse agonist LEAP2 may reveal a potential therapeutic target for ghrelin-related diseases, including type 2 diabetes and obesity, due to its reciprocal relationship with ghrelin[4] and elevated expression levels following RYGB.[7]

The administration of ghrelin in rodents and humans stimulates food intake and increases plasma glucose.[5,12,13] Several groups have investigated the effect of exogenous LEAP2 on food intake and plasma glucose in rodents. A pioneering study demonstrated lower food intake in LEAP2-treated compared with vehicle-treated mice;[5] however, this finding could not be confirmed in succeeding studies.[6,7,14] Nevertheless, LEAP2 treatment has consistently been shown to impair ghrelin-induced food intake and hyperglycemia in rodents.[5,6,14–16] Notably, only two studies besides the study of Ge et al. have demonstrated effects of LEAP2 administration alone, i.e., a decrease in plasma glucose after intraperitoneal LEAP2 administration in mice[17] and reduced binge-like eating after central administration of a truncated LEAP2 form in mice.[18] Whether exogenous LEAP2 affects food intake and glucose metabolism in humans has not been investigated.

In the present study, we studied the effects of exogenous LEAP2 in healthy men and show that it lowers postprandial glucose excursions and suppresses food intake (effects that may be mediated via the GHSR as informed by experiments in GHSR-null mice). Whether the striking effects of exogenous LEAP2 in humans will revitalize the GHSR as a therapeutic target in metabolic diseases awaits further studies.

RESULTS

Intravenous infusion of LEAP2 results in supraphysiological steady-state plasma concentrations in lean men

To investigate the metabolic effects of LEAP2 in humans, we carried out an intravenous infusion of LEAP2 (~25 pmol/kg/min), resulting in supraphysiological plasma concentrations in 20 lean, young men (see Table 1 for baseline characteristics and Figure 1 for an overview of the randomized, double-blind, placebo-controlled study design). Using an in-house radioimmunoassay directed against the N-terminal part of LEAP2, we determined LEAP2 plasma concentrations during LEAP2 and placebo infusions. During LEAP2 and placebo infusions, the baseline concentrations of LEAP2 plasma concentrations were similar (Figure 2A). During LEAP2 infusion, plasma concentrations of LEAP2 reached steady state after 45 min of infusion with a mean concentration of 41.2 ± 1.1 ng/mL (Figure 2A), ~2.6-fold higher than the mean plasma LEAP2 concentration during placebo infusion (Figure 2A). During placebo infusion, no change in LEAP2 concentration was found in response to the liquid mixed meal (Figure 2A), suggesting that endogenous LEAP2 concentrations are not acutely affected by food intake in lean, young men. The participants reported no adverse events during the infusions.

Exogenous LEAP2 alters the postprandial plasma glucose response

First, we assessed the effect of exogenous LEAP2 on postprandial glucose metabolism in healthy men. For this purpose, participants were given a standardized liquid mixed meal (65 min after infusion start). During LEAP2 infusion, the postprandial plasma glucose peaks were lower than during placebo infusion (Figure 2B; Table 2). Furthermore, the area under the curve (AUC) for the entire infusion period ($AUC_{0–255\ min}$) and the postprandial AUC ($AUC_{60–255\ min}$) were lower during LEAP2 infusion compared with placebo (Table 2), demonstrating a reduced postprandial glucose response during LEAP2 infusion.

Insulinotropic and anti-lipolytic effects of exogenous LEAP2 during fasting

We measured plasma concentrations of insulin, C-peptide, glucagon, and calculated C-peptide/glucose ratio—the latter as a measure of pancreatic beta cell secretion—before and after the liquid mixed meal test (i.e., during fasting and postprandial conditions) in the healthy, young men. In the fasting state, the circulating concentrations of insulin and C-peptide and C-peptide/glucose ratio were higher during LEAP2 infusion compared with placebo, as assessed by $AUC_{0–60\ min}$ (Figures 2C–2E; Table 2). Accordingly, postprandial baseline values (i.e., at the end of the fasting period, time = 60 min) were higher for both insulin, C-peptide, and C-peptide/glucose ratio (Figures 2C–2E; Table 2). The insulinotropic effect of LEAP2 during fasting was supported by a shorter time to peak for C-peptide during LEAP2 infusion (Table 2). An increase of ~2 pmol/L in glucagon concentrations was observed both in the fasting state and postprandially during LEAP2 infusion (Figure 2F; Table 2). Plasma concentrations of free fatty acids, glycerol, and triglycerides were measured during fasting and postprandial conditions (Figures 2G–2I; Table 2). In the

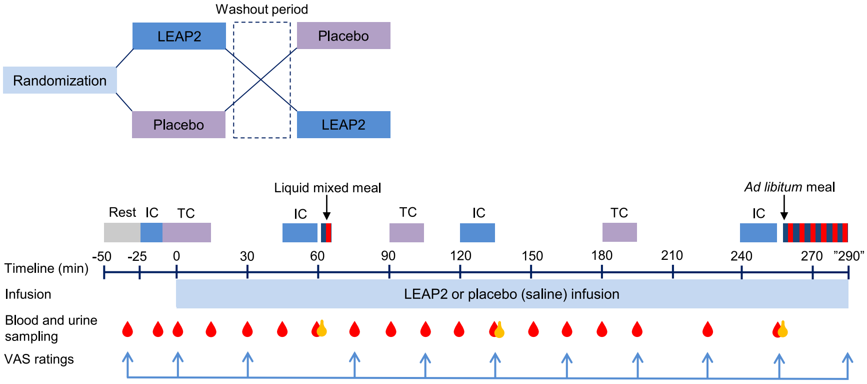

Figure 1. Overview of the clinical study design

The study was designed as a randomized, double-blind, placebo-controlled, crossover study including 20 healthy, young men. IC, indirect calorimetry; LEAP2, liver-expressed antimicrobial peptide 2; TC, thermal imaging camera; VAS, visual analog scale.

fasting state, circulating glycerol ($AUC_{0–60\ min}$) concentrations were lower during LEAP2 infusion compared with placebo infusion (Figure 2H; Table 2), suggesting a decreased lipolytic activity. No differences in $AUC_{0–60\ min}$ values were found for free fatty acids or triglycerides, but postprandial baseline concentrations (time = 60 min) of free fatty acids and glycerol were lower during LEAP2 infusion (Table 2).

Exogenous LEAP2 does not alter gastric emptying

Because ghrelin is known to increase the gastric emptying rate,[19] we admixed acetaminophen to the liquid mixed meal in order to evaluate postprandial plasma excursions of acetaminophen as an indirect marker of gastric emptying, as previously validated.[20] Peak plasma acetaminophen concentrations and time to peak plasma acetaminophen concentrations revealed no difference between LEAP2 and placebo infusions (Figure 2J; Table 2), suggesting that exogenous LEAP2 does not alter gastric emptying rate of a liquid mixed meal.

Exogenous LEAP2 decreases plasma concentrations of GH, but not ghrelin

GH release reflects activity of the GHSR and is stimulated by acute administration of ghrelin.[21] To test whether exogenous LEAP2 reduces the production or secretion of GH and ghrelin, we measured plasma concentrations of both hormones (Figures 2K and 2L; Table 2). Notably, GH concentrations were suppressed during LEAP2 infusion compared with placebo, as assessed by AUC (Table 2). When assessing the fasting ($AUC_{0–60\ min}$) and postprandial ($AUC_{60–255\ min}$) periods separately, only the postprandial period was lower during LEAP2 infusion compared with placebo (Table 2), suggesting that LEAP2 mainly suppresses GH postprandially. The postprandial GH suppression was supported by a lower nadir during LEAP2 infusion compared with placebo infusion (Table 2). Plasma concentrations of ghrelin (measured as active acylated ghrelin) were unaffected by LEAP2 infusion (Table 2); hence, the metabolic effects of exogenous LEAP2 in healthy men do not seem to be a result of altered plasma concentrations of ghrelin. Together, these results support previous findings on suppression of ghrelin-induced GH release in a dose-dependent manner in rodents[5,14] and suggest that LEAP2 may reduce GH release independently of plasma concentrations of ghrelin in humans.

No effect of LEAP2 on sensations of hunger, satiety, prospective food consumption, fullness, nausea, comfort, and thirst

As ghrelin infusion in humans has been reported to increase appetite sensations,[13] we investigated the effect of LEAP2 on several appetite-related sensations using visual analog scale (VAS) ratings. We found no differences in VAS ratings of hunger, satiety, prospective food consumption, fullness, nausea, comfort, or thirst from experimental days with LEAP2 and placebo infusions (Figures 3A–3G). Moreover, we found no differences between LEAP2 and placebo infusions when comparing the VAS ratings given just before (time = 255 min) as well as just after (time ~272 min) the *ad libitum* meal. Thus, changes in VAS ratings of hunger, prospective food consumption, or nausea could not explain the reduced food intake observed during LEAP2 infusion. Importantly, VAS ratings indicated a low level of nausea and a high level of comfort throughout both study days (Figures 3E and 3F).

Exogenous LEAP2 does not affect resting energy expenditure, utilization of metabolic substrates, or brown adipose tissue thermogenesis

Since ghrelin has been proposed as a regulator of systemic energy expenditure,[1] we investigated the effect of exogenous LEAP2 on resting energy expenditure, respiratory quotient, and protein turnover in healthy individuals assessed by indirect calorimetry and renal urea excretion, respectively. Using this approach, we found no differences in resting energy expenditure or respiratory quotient between LEAP2 and placebo infusions (Figure 3H; respiratory quotient data not shown). Moreover, we found no difference in renal urea excretion at the two consecutive time points or total urea excretion between study days (data not shown). As ghrelin has been suggested to suppress brown adipose tissue (BAT) activity in rodents,[1] we also investigated BAT thermogenesis using a thermal imaging camera measuring temperatures of the skin above the two supraclavicular BAT depots and over the manubrium of the sternum; the latter as a control reflecting overall cutaneous circulation. We found no differences in BAT thermogenesis between LEAP2 and placebo infusions calculated as the average of the supraclavicular skin temperatures subtracting the manubrium skin temperature (Figure 3I), indicating that LEAP2 does not affect BAT thermogenesis in humans.

Exogenous LEAP2 reduces food intake during an *ad libitum* meal

Next, food intake was assessed by an *ad libitum* meal test at the end of the infusion period. Here, we found an ~12% reduction in

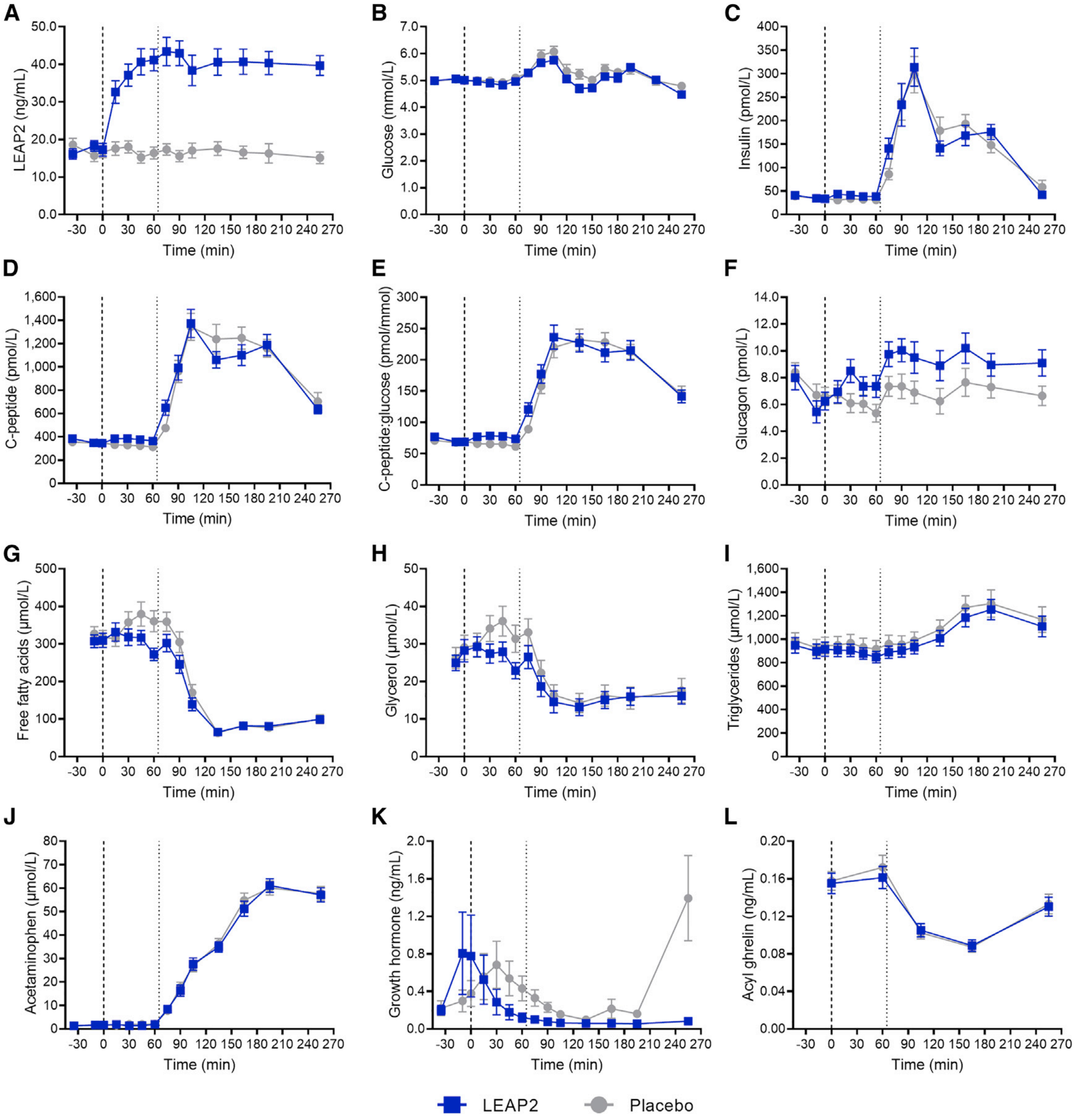

Figure 2. LEAP2 alters postprandial plasma concentrations of glucose, glucagon, and growth hormone during a liquid mixed meal test and fasting plasma concentrations of insulin, C-peptide, C-peptide/glucose ratio, glucagon, and glycerol in healthy, young men

Plasma concentrations of LEAP2 (A), glucose (B), insulin (C), C-peptide (D), C-peptide/glucose ratio (E), glucagon (F), free fatty acids (G), glycerol (H), triglycerides (I), acetaminophen (J), growth hormone (K), and acyl ghrelin (L). Bold dotted line, infusion start (0 min); thin dotted line, liquid mixed meal test (65 min); blue square symbols, LEAP2 infusion; gray round symbols, placebo infusion (n = 20). Data are presented as mean ± SEM. Student's paired t test of $AUC_{60–255\ min}$: $p = 0.017$ (B), $p < 0.001$ (F), and $p < 0.001$ (K). Student's paired t test of $AUC_{0–60\ min}$: $p < 0.001$ (C), $p = 0.018$ (D), $p = 0.003$ (E), $p = 0.038$ (F), and $p = 0.039$ (H). AUC, area under the curve.

energy intake in the healthy, young men during LEAP2 infusion compared with placebo demonstrated by reduced total caloric intake and caloric intake per kg body weight (Figures 4A and 4B). In addition to reduced food intake, a shorter meal duration during LEAP2 infusion was also observed (Figure 4C).

GHSR-dependent effect of LEAP2 dosing on plasma glucose and food intake in mice

We evaluated the effects of a single subcutaneous dose of LEAP2 on glucose metabolism and food intake in GHSR-null mice and wild-type littermates to tease out GHSR-dependent effects. In line with the observed effects in humans, LEAP2 dosing significantly diminished increases in plasma glucose during *ad libitum* food intake 1 h after dosing in wild-type mice as compared with vehicle (Figure 5A). In contrast, a similar dose of LEAP2 did not affect plasma glucose in GHSR-null mice (Figure 5A), indicating that the glucose-lowering effect of LEAP2 in the postprandial state may be mediated through the GHSR. Of note, the glucose-lowering effect of LEAP2 was only observed after 1 h and not at the later time points (Figure 4A). In line with the human

CellPress
OPEN ACCESS

Table 2. Overview of plasma measurements in the clinical study

	LEAP2	Placebo (saline)	p value (paired t test)
**Glucose**			
Baseline (mmol/L)	5.02 ± 0.06	5.01 ± 0.07	0.900
Peak (mmol/L)	6.21 ± 0.12	6.58 ± 0.13	0.005
Time to peak (min)	140 ± 12.7	137 ± 10.7	0.808
Postprandial baseline (mmol/L)	4.96 ± 0.05	5.10 ± 0.08	0.077
Postprandial end (mmol/L)	4.48 ± 0.11	4.75 ± 0.12	0.056
AUC (mmol/L × min)	1,288 ± 13.4	1,336 ± 18.5	0.013
–60 min (mmol/L × min)	295 ± 3.02	300 ± 3.67	0.198
$AUC_{60–255\ min}$ (mmol/L × min)	992 ± 12.4	1,037 ± 16.9	0.017
**Insulin**			
Baseline (pmol/L)	36.4 ± 1.70	36.3 ± 2.08	0.984
Peak (pmol/L)	347 ± 38.6	356 ± 35.4	0.734
Time to peak (min)	110 ± 6.28	120 ± 9.23	0.433
Postprandial baseline (pmol/L)	38.6 ± 2.09	31.1 ± 2.08	0.017
AUC (nmol/L × min)	33.8 ± 2.63	33.3 ± 2.34	0.775
$AUC_{0–60\ min}$ (nmol/L × min)	2.39 ± 0.09	1.92 ± 0.13	<0.001
$AUC_{60–255\ min}$ (nmol/L × min)	31.5 ± 2.60	31.4 ± 2.28	0.985
**C-peptide**			
Baseline (pmol/L)	360 ± 21.4	350 ± 15.8	0.673
Peak (pmol/L)	1,524 ± 106	1,647 ± 125	0.317
Time to peak (min)	136 ± 9.01	160 ± 11.2	0.028
Postprandial baseline (pmol/L)	365 ± 20.2	313 ± 15.9	0.010
AUC (nmol/L × min)	218 ± 11.7	222 ± 11.5	0.697
$AUC_{0–60\ min}$ (nmol/L × min)	22.5 ± 1.18	19.7 ± 1.00	0.018
$AUC_{60–255\ min}$ (nmol/L × min)	196 ± 11.2	202 ± 10.9	0.490
**C-peptide/glucose ratio**			
Baseline (pmol/mmol)	71.4 ± 3.93	69.8 ± 2.82	0.675
Peak (pmol/mmol)	269 ± 17.9	268 ± 17.6	0.959
Time to peak (min)	140 ± 9.24	153 ± 10.3	0.179
Postprandial baseline (pmol/mmol)	73.6 ± 4.03	61.4 ± 3.03	0.002
AUC (nmol/mmol × min)	41.4 ± 2.34	40.8 ± 1.89	0.705
$AUC_{0–60\ min}$ (nmol/mmol × min)	4.57 ± 0.23	3.92 ± 0.18	0.003
$AUC_{60–255\ min}$ (nmol/mmol × min)	36.8 ± 2.23	36.8 ± 1.78	1.000
**Glucagon**			
Baseline (pmol/L)	6.57 ± 0.76	7.23 ± 0.68	0.237
Peak (pmol/L)	13.0 ± 0.94	11.2 ± 0.72	0.003
Time to peak (min)	116 ± 12.5	117 ± 17.1	0.926
Postprandial baseline (pmol/L)	7.35 ± 0.82	5.35 ± 0.67	0.005
AUC (nmol/L × min)	2.26 ± 0.20	1.74 ± 0.14	<0.001
$AUC_{0–60\ min}$ (nmol/L × min)	0.446 ± 0.044	0.375 ± 0.035	0.038
$AUC_{60–255\ min}$ (nmol/L × min)	1.82 ± 0.17	1.36 ± 0.13	<0.001
**Free fatty acids**			
Baseline (μmol/L)	339 ± 18.4	361 ± 20.9	0.419
Nadir (μmol/L)	58.4 ± 3.94	57.7 ± 3.70	0.860
Time to nadir (min)	155 ± 7.63	168 ± 11.1	0.317
Postprandial baseline (μmol/L)	272 ± 16.4	360 ± 28.0	0.013
AUC (mmol/L × min)	43.2 ± 2.49	48.1 ± 2.74	0.171

(*Continued on next page*)

Table 2. *Continued*

	LEAP2	Placebo (saline)	p value (paired t test)
$AUC_{0–60\ min}$ (mmol/L × min)	18.9 ± 1.18	20.9 ± 1.43	0.258
$AUC_{60–255\ min}$ (mmol/L × min)	24.4 ± 1.51	27.2 ± 1.54	0.150
Glycerol			
Baseline (μmol/L)	26.6 ± 2.45	28.4 ± 3.12	0.379
Nadir (μmol/L)	11.7 ± 2.12	12.1 ± 2.44	0.761
Time to nadir (min)	144 ± 10.2	164 ± 11.8	0.201
Postprandial baseline (μmol/L)	22.9 ± 2.12	31.4 ± 3.65	0.007
AUC (mmol/L × min)	4.87 ± 0.57	5.49 ± 0.70	0.080
$AUC_{0–60\ min}$ (mmol/L × min)	1.65 ± 0.15	1.95 ± 0.19	0.039
$AUC_{60–255\ min}$ (mmol/L × min)	3.22 ± 0.44	3.54 ± 0.54	0.211
Triglycerides			
Baseline (μmol/L)	920 ± 62.2	953 ± 70.4	0.539
Peak (μmol/L)	1,275 ± 84.8	1,353 ± 113	0.279
Time to peak (min)	195 ± 5.33	192 ± 6.85	0.725
Postprandial baseline (μmol/L)	847 ± 48.3	916 ± 73.2	0.159
AUC (mmol/L × min)	263 ± 16.7	279 ± 22.1	0.251
$AUC_{0–60\ min}$ (mmol/L × min)	53.5 ± 3.14	56.8 ± 4.41	0.284
$AUC_{60–255\ min}$ (mmol/L × min)	210 ± 13.8	222 ± 17.9	0.258
Growth hormone			
Baseline (ng/mL)	0.594 ± 0.300	0.299 ± 0.106	0.372
Nadir (ng/mL)	0.042 ± 0.004	0.084 ± 0.012	0.001
Time to nadir (min)	116 ± 17.2	128 ± 13.9	0.592
Postprandial baseline (ng/mL)	0.121 ± 0.048	0.432 ± 0.133	0.014
AUC (ng/mL × min)	33.8 ± 11.8	106 ± 18.3	0.001
$AUC_{0–60\ min}$ (ng/mL × min)	20.2 ± 9.76	32.0 ± 9.57	0.312
$AUC_{60–255\ min}$ (ng/mL × min)	13.6 ± 2.32	73.7 ± 14.5	<0.001
Acyl ghrelin			
Baseline (ng/mL)	0.155 ± 0.011	0.158 ± 0.010	0.730
Nadir (ng/mL)	0.088 ± 0.006	0.084 ± 0.004	0.343
Postprandial baseline (ng/mL)	0.161 ± 0.012	0.172 ± 0.013	0.264
AUC (ng/mL × min)	31.2 ± 1.94	31.7 ± 1.96	0.634
$AUC_{0–60\ min}$ (ng/mL × min)	9.50 ± 0.67	9.90 ± 0.65	0.387
$AUC_{60–255\ min}$ (ng/mL × min)	21.7 ± 1.33	21.8 ± 1.34	0.875
Acetaminophen			
Peak (μmol/L)	65.1 ± 2.52	66.3 ± 2.75	0.548
Time to peak (min)	209 ± 6.34	215 ± 7.93	0.297

Data are presented as mean ± SEM. AUC, area under the curve; LEAP2, liver-expressed antimicrobial peptide 2.

results, LEAP2 dosing lowered the cumulative food intake in wild-type mice 2 and 4 h after dosing (Figure 5B), but no effect of LEAP2 was found in GHSR-null mice (Figure 5B). Together, these results suggest that the GHSR is a prerequisite for the observed metabolic effects of LEAP2 in mice and provide *in vivo* insights into LEAP2 as a GHSR ligand with opposing actions to the orexigenic hormone ghrelin. In a separate cohort of fasted female wild-type mice, we observed no effect of exogenous LEAP2 on fasting insulin concentrations following LEAP2 dosing (Figure 5C), contrasting our human data. As in humans, LEAP2 did not affect fasting plasma glucose concentrations in wild-type mice either (Figure 5D).

Pharmacokinetic parameters of exogenous LEAP2 in healthy men

In order to describe the kinetics of exogenous LEAP2, 8 of the 20 study participants were subjected to a separate intravenous infusion of LEAP2 reaching supraphysiological steady-state plasma concentrations followed by a 150-min wash-out period (Figure S1A). Based on a pharmacokinetic model taking baseline plasma concentrations of LEAP2 into account,[22] we estimated the volume of distribution of LEAP2 to 14 ± 5.3 L, metabolic clearance rate of LEAP2 to 0.86 ± 0.17 L/min, half-life of LEAP2 to 9.1 ± 2.5 min, and endogenous production rate of LEAP2 to 1.9 ± 0.14 nmol/min. Taken

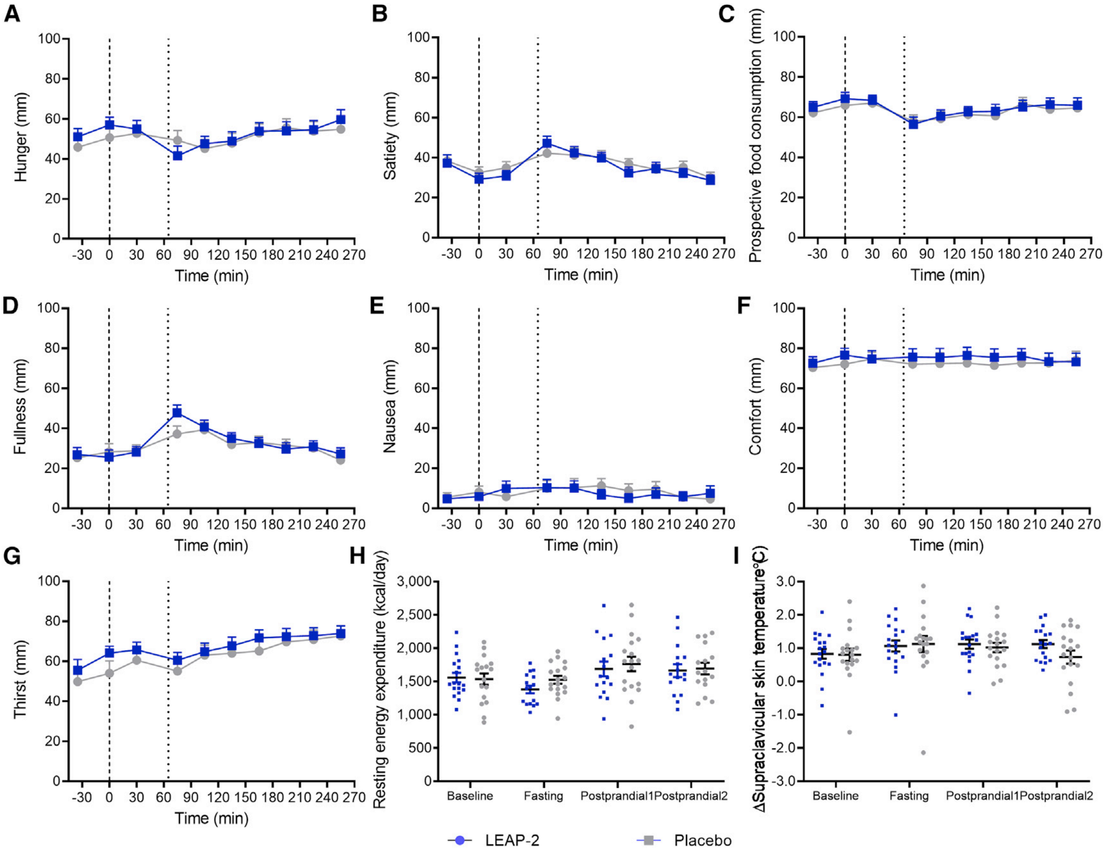

Figure 3. No effect on ratings of hunger and other sensations, resting energy expenditure, or brown adipose tissue thermogenesis during LEAP2 infusion in healthy, young men

(A–G) Visual analog scale ratings of hunger (A), satiety (B), prospective food consumption (C), fullness (D), nausea (E), comfort (F), and thirst (G).

(H) Resting energy expenditure.

(I) Manubrium-corrected supraclavicular skin temperature. Bold dotted line, infusion start (0 min); thin dotted line, liquid mixed meal test (65 min); blue square symbols, LEAP2 infusion; gray round symbols, placebo infusion (n = 20).

Data are presented as mean ± SEM. No significant differences (Student's paired t test of area under the curve, [A–G]; repeated-measures mixed effects model, [H and I]).

together, these findings indicate that LEAP2 is rapidly cleared from the circulation. This finding is in agreement with previous findings in mice subjected to a single intravenous dose of LEAP2.[5]

DISCUSSION

LEAP2 was recently discovered as an endogenous circulating hormone in rodents with antagonistic effects on ghrelin actions and inverse effects on GHSR constitutive activity.[5,6] Here, we demonstrate that exogenous LEAP2 reduces postprandial glucose excursions and suppresses food intake in healthy men. In addition, we report a lower glucose peak and food intake in wild-type mice, but not in GHSR-null mice, after LEAP2 administration, suggesting that the plasma glucose- and appetite-lowering effects of LEAP2 are mediated by the ghrelin receptor, GHSR, in mice. The observed effects on both glucose homeostasis and appetite regulation may revitalize the GHSR as therapeutic target in metabolic diseases in the future.

A pioneering study characterizing the pharmacological effects of LEAP2 in rodents[5] demonstrated a lower food intake in mice treated with LEAP2 compared with vehicle-treated mice. Using a randomized, double-blind, placebo-controlled crossover design, we now demonstrate that exogenous LEAP2 reduces food intake during an *ad libitum* meal test (of pasta Bolognese) in healthy men. On average, participants consumed 432 kJ less during LEAP2 infusion compared with placebo (corresponding to an ~12% reduction) and spent less time eating before feeling comfortably full. For comparison, 432 kJ corresponds approximately to the energy content in a 25 cL soft drink. Importantly, this anorectic effect was observed without a concomitant increase in nausea. A sustained reduction of 432 kJ per meal for longer periods would translate into robust weight loss, but it remains to be determined whether the effect is reproducible in overweight individuals and whether it is sustained with prolonged dosing of LEAP2 and whether the response can be optimized by altering the LEAP2 dosage. For perspective, acute GLP-1 infusion (inducing an ~8-fold increase in plasma

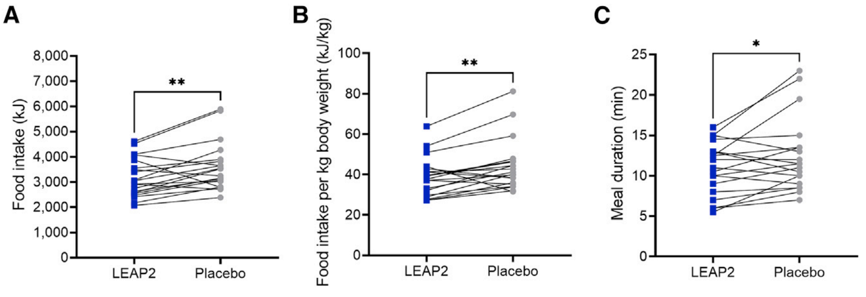

Figure 4. LEAP2 reduces food intake during an *ad libitum* meal test in healthy, young men

Ad libitum food intake assessed by total intake (A), intake per kg body weight (B), and duration of the *ad libitum* meal (C). Blue square symbols, LEAP2 infusion; gray round symbols, placebo infusion (n = 20). Data are presented as mean ± SEM. $^{*}p < 0.05$; $^{**}p < 0.01$ (Student's paired t test). Mean differences 432 (95% confidence interval [CI] 155–709) kJ, p = 0.0041 (A); 5.4 (95% CI 2.1–8.7) kJ/kg, p = 0.0029 (B); 1.5 (95% CI 0.2–2.9) min, p = 0.0303 (C).

concentration of GLP-1) in healthy, normal-weight, young men resulted in a similar reduction in energy intake of ~12% during a comparable *ad libitum* meal test.[23]

Interestingly, the appetite-suppressing effect of LEAP2 was reproduced in wild-type mice, but not in GHSR-null mice, showing that the GHSR is required for the reduced food intake observed in LEAP2-treated mice. Our data confirm the finding by Ge et al.[5] demonstrating a lower food intake in LEAP2-treated compared with vehicle-treated mice, in contrast to a number of other studies.[6,7,14] The inconsistent finding on LEAP2-induced modulation of food intake may be related to the level of stress in the studied animals, as stress hormones strongly induce ghrelin secretion. In our study designed to measure plasma glucose, we expect high level of stress and accordingly high plasma ghrelin concentrations. However, the fact that LEAP2 administration consistently has been shown to reverse ghrelin-induced food intake[5,6,14,16] makes it conceivable that LEAP2 mainly acts as a competitive antagonist impairing ghrelin-induced activation of GHSR in rodents. This is further supported by a recent study indicating that LEAP2 deletion in mice enhances the actions of ghrelin.[15] Of note, it remains to be explored whether exogenous LEAP2 reduces food intake in animal models of impaired metabolic control, such as diet-induced obesity.

The insulinotropic properties of the LEAP2 family of peptide hormones *in vitro*[6,7] prompted us to investigate the effect of exogenous LEAP2 on postprandial glucose metabolism in healthy humans. Here, we report reduced postprandial glucose excursions following a standardized liquid mixed meal during LEAP2 infusion. Plasma profiles of insulin and C-peptide revealed an insulinotropic effect of LEAP2 during fasting, but not postprandially. A concomitant increase in the C-peptide/glucose ratio during fasting indicates that LEAP2 may increase the β cell sensitivity to glucose in the fasted state without enhancing glucose-stimulated insulin secretion after meal ingestion, which is in agreement with our recent findings for the endogenous LEAP2 fragment, $LEAP2_{38–47}$.[7] The mechanisms underlying the reduced postprandial plasma glucose excursions observed in the present study remain uncertain, but we speculate that LEAP2 may act through brain-mediated regulation of hepatic glucose production, as seen with insulin,[24] or increase peripheral insulin sensitivity, the latter possibly as an indirect effect to decreased plasma concentrations of GH.[25] Moreover, we cannot rule out that the increased insulin concentrations observed during LEAP2 infusion in the fasting state may prime insulin-sensitive tissues before meal ingestion. Gastric emptying was not altered by exogenous LEAP2, as previously reported for ghrelin,[19] suggesting that the lower postprandial plasma glucose peak observed during LEAP2 infusion is not a result of delayed gastric emptying of the meal.

We observed a small increment in plasma glucagon concentrations during LEAP2 infusion. The increase in plasma glucagon concentration may counteract the glucose-lowering effect of exogenous LEAP2. The GHSR has been localized to both α and β cells as well as somatostatin-producing δ cells and pancreatic polypeptide cells in the human and mouse pancreas.[17,26–28] It may be of interest, therefore, to investigate whether a GHSR-dependent glucoregulatory effect of LEAP2 is affected by the intra-islet communication that is thought to exist between the different GHSR-expressing pancreatic cell types.[26,29]

The LEAP2-induced reduction of postprandial plasma glucose excursions was reproducible in wild-type mice, but not in GHSR-null mice, when measuring plasma glucose 1 h after dosing. This suggests that the glucose-lowering effect of LEAP2 in mice is dependent on the GHSR. It should be noted that three studies have already investigated the effect of exogenous LEAP2 on glucose metabolism in rodents: (1) in a recent study in rats,[14] it was demonstrated that centrally injected LEAP2 suppresses the hyperglycemic effect of centrally injected ghrelin; (2) a decrease in plasma glucose following intraperitoneally administered LEAP2 in mice was recently reported;[17] and (3) in male NMRI mice, LEAP2 did not affect the blood glucose during an oral glucose tolerance test.[7] Taken together, these results suggest that any glucose-lowering effect of LEAP2 might depend on several factors, including way of LEAP2 administration, nature of the glycemia, and the phenotype of the organism investigated.

During fasting, GH promotes lipid mobilization and oxidation as a means of fat utilization.[25] Exogenous ghrelin has been reported to induce lipolysis in humans independently of GH,[30] mirroring the physiological effect of endogenous ghrelin and GH during fasting.[1] This prompted us to investigate the effect of exogenous LEAP2 on plasma concentrations of triglycerides, free fatty acids, and glycerol—the substrate and products, respectively, of lipolysis—in fasting and postprandial states. Compared with placebo, LEAP2 infusion decreased glycerol in the fasted state ($AUC_{0–60\ min}$) and plasma concentrations of both glycerol and free fatty acids were lower preprandially (at time point 60 min), suggesting inhibition of lipolysis during LEAP2 infusion. It remains to be established whether LEAP2 has any anti-lipolytic effect or whether lipolysis may be indirectly mediated via effects on insulin secretion and/or GH secretion.

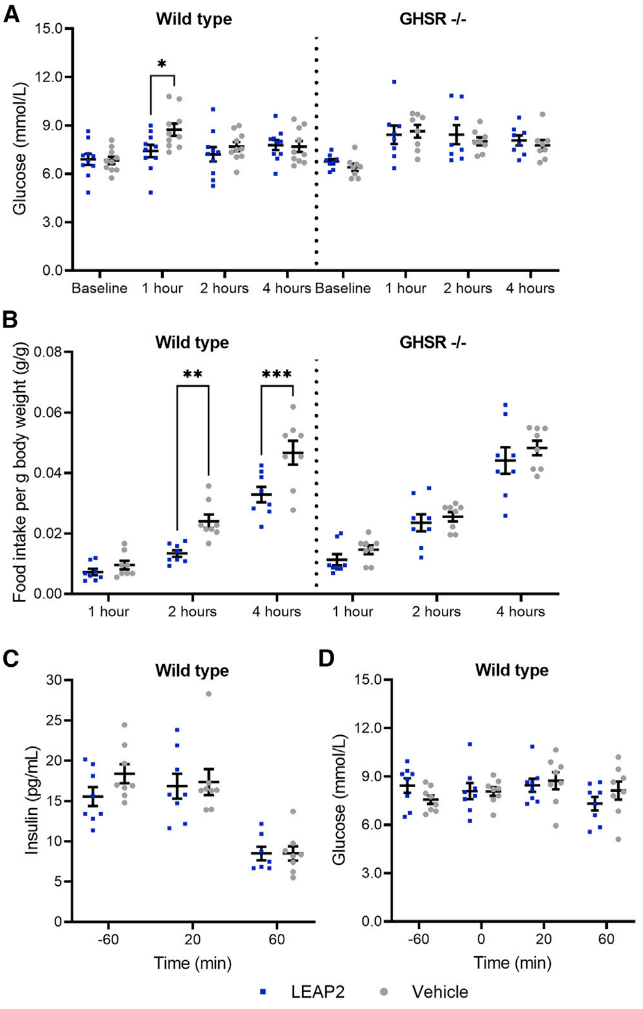

Figure 5. LEAP2 reduces postprandial plasma glucose and food intake in mice in a GHSR-dependent manner

(A and B) Plasma glucose concentrations in wild-type (mean body weight = 22.3 g) and GHSR-null (GHSR$^{-/-}$) mice (mean body weight = 22.3 g) at baseline and 1, 2, and 4 h after LEAP2 or vehicle dosing (A) and cumulative food intake 1, 2, and 4 h after dosing (B).

(C and D) Fasting insulin (C) and plasma glucose (D) concentrations in wild-type mice (mean body weight = 22.5 g). Blue square symbols, LEAP2 dosing; gray round symbols, vehicle dosing (n = 8–10). Data are presented as mean ± SEM. Repeated-measures two-way ANOVA: p = 0.0007 (B). *p < 0.05; **p < 0.01; ***p < 0.001 (Šidák's *post hoc* test). GHSR, growth hormone secretagogue receptor.

Because ghrelin has been proposed to modulate systemic energy expenditure,[1] we tested whether exogenous LEAP2 could have the opposite effect on resting energy expenditure, substrate utilization, and BAT thermogenesis. We observed no effect on resting energy expenditure or utilization of carbohydrates, fat, or protein—the latter assessed by respiratory quotient and renal urea excretion. Moreover, no effect on BAT thermogenesis was found. Limitations of these measurements should be considered. Indirect calorimetry depends on the participant's ability to rest, and in 23 tests out of 160, the readings did not reach steady state, probably due to the other study procedures and therefore limited test time. Thermal imaging of skin temperatures depends on other factors than BAT thermogenesis, such as the surrounding temperature. To correct for the overall cutaneous heat production in the air-conditioned room, we assessed BAT thermogenesis by manubrium-corrected supraclavicular skin temperatures. Of note, this study was powered to detect differences in food intake, and thus, regarding the secondary endpoints, our study might be underpowered.

We measured plasma concentrations of GH as a sign of GHSR activation, since this is the most sensitive physiological response to GHSR activation.[5] Plasma concentration of GH was lower during LEAP2 infusion compared with placebo, indicating reduced GHSR activity. Interestingly, plasma profiles of ghrelin did not differ between LEAP2 and placebo infusions, pointing to absence of feedback regulation of ghrelin secretion, known to occur for many other hormonal systems. Taken together, our data confirm previous *in vitro* findings that LEAP2 downregulates GHSR activity independently of ghrelin concentrations,[4,6,7] thus suggesting that the metabolic effects of exogenous LEAP2 observed in humans are mediated—at least partly—through GHSR. Whether the decrease in plasma concentration of GH observed postprandially in this study is involved in the reduction of the postprandial glucose excursions remains to be explored. As previously speculated, exogenous LEAP2 does not seem to inhibit the production or secretion of ghrelin.[5] The plasma profile of GH during placebo infusion followed a pattern similar to that of ghrelin, which is consistent with the known physiological regulation according to energy status,[31] i.e., increased concentrations during fasting, decreased postprandial concentrations, and returning to fasting concentrations at the end of the postprandial period. The pulsatile pattern of GH secretion may explain the large variation observed in the dataset.[32] Importantly, both GH and ghrelin plasma concentrations were within physiological ranges reported previously.[31]

Until now, the effects of LEAP2 exposure have not been investigated in humans. Importantly, no adverse reactions were observed during the infusions. LEAP2 infusion resulted in a 2.6-fold steady-state concentration as compared with placebo infusion, reaching supraphysiological concentrations as compared with previous plasma measurements in lean individuals.[4] Plasma concentrations of LEAP2 during placebo infusion and baseline were within concentration ranges reported previously,[4,7] suggesting that LEAP2 is an endogenous peptide measured in the nanomolar range. Moreover, our data confirm that endogenous LEAP2 assessed during placebo infusion was not affected by a liquid mixed meal test in lean individuals in agreement with previous findings in lean individuals.[4] Whether endogenous LEAP2 concentrations change with food intake in overweight individuals is presently uncertain due to contrasting findings.[4,7] Of notice, we cannot rule out that the observed effects of exogenous LEAP2 would have been more pronounced employing a higher infusion rate of LEAP2, and future studies will, therefore, be required to investigate a possible dose-response relationship. The pharmacokinetic model of exogenous LEAP2 shows a half-life of LEAP2 amounting to ~9 min; thus, an extension of the half-life is necessary—for example, through lipidation—if LEAP2 should have a pharmacological potential in the future.

Limitations of the study

Plasma concentrations of insulin-like growth factor 1 were not measured. Also, the study was designed to investigate the effects of exogenous LEAP2 in healthy men and was not designed to answer mechanistic effects of exogenous LEAP2 in humans. Since only one dose of LEAP2 was used, the dose-response relationship remains to be investigated.

In conclusion, we demonstrate that exogenous LEAP2 resulting in supraphysiological plasma concentrations reduces postprandial glucose excursions and *ad libitum* food intake in young, healthy men. These effects were also observed in wild-type mice, but not in GHSR-null mice, suggesting that the glucose-lowering and appetite-suppressing effects of LEAP2—at least partly—depend on the GHSR in mice. Together, these findings raise the possibility that LEAP2 might constitute a clinically relevant pharmacological target for the treatment of obesity.

STAR★METHODS

Detailed methods are provided in the online version of this paper and include the following:

- KEY RESOURCES TABLE
- RESOURCE AVAILABILITY
  - Lead contact
  - Materials availability
  - Data and code availability
- EXPERIMENTAL MODEL AND SUBJECT DETAILS
  - Human participants
  - Ethics approval
  - Mouse models
- METHOD DETAILS
  - Peptides and preparation of infusions
  - Clinical study design and experimental procedures
  - Visual analog scale ratings
  - Indirect calorimetry
  - Thermal imaging
  - Urine sampling
  - Human plasma analyses
  - Pharmacokinetic parameters of LEAP2 in healthy men
  - Effects of LEAP2 on glucose metabolism and food intake in mice
  - Effects of LEAP2 on insulin and plasma glucose concentrations in fasted mice
- QUANTIFICATION AND STATISTICAL ANALYSIS

SUPPLEMENTAL INFORMATION

Supplemental information can be found online at https://doi.org/10.1016/j.xcrm.2022.100582.

ACKNOWLEDGMENTS

We thank the participants for their commitment to the clinical study; K.H. Rasmussen, D.B. Nielsen, A.H. Klitgaard, and J. Bach for laboratory assistance; and A.M. Ellegaard for critical reading of the manuscript. C.A.H. and S.H. received research grants from the Innovation Fund Denmark (grant number 8053-00026B) and the Danish Diabetes Academy, respectively. The latter is funded by the Novo Nordisk Foundation (grant number NNF17SA0031406). Synthetic LEAP2 used for animal studies was a gift from Novo Nordisk (Bagsværd, Denmark). The graphical abstract was created with BioRender.com.

AUTHOR CONTRIBUTIONS

Conceptualization, C.A.H. and F.K.K.; methodology, C.A.H., L.S.G., M.B.C., B. Holst, and F.K.K.; investigation, C.A.H., M.S.J., and S.H.; resources, C.A.H., S.H., S.B., B. Hartmann, F.D., and J.J.H.; writing – original draft, C.A.H. and S.H.; writing – review & editing, C.A.H., M.S.J., S.H., L.S.G., S.B., K.S.-J., B. Hartmann, J.J.H., F.D., T.V., M.B.C., B. Holst, and F.K.K.; supervision, L.S.G., M.B.C., T.V., B. Holst, and F.K.K.; funding acquisition, C.A.H., B. Holst, and F.K.K.

DECLARATION OF INTERESTS

F.K.K. has served on scientific advisory panels and/or been part of speaker's bureaus for, served as a consultant to, and/or received research support from Amgen, AstraZeneca, Boehringer Ingelheim, Carmot Therapeutics, Eli Lilly, Gubra, MedImmune, MSD/Merck, Mundipharma, Norgine, Novo Nordisk, Sanofi, and Zealand Pharma. L.S.G. is in speaker's bureau for Eli Lilly. T.V. has served on scientific advisory panels for, been part of speaker's bureaus for, served as a consultant to, and/or received research support from Amgen, AstraZeneca, Boehringer Ingelheim, Eli Lilly, Gilead, Mundipharma, MSD/Merck, Novo Nordisk, Sanofi, and Sun Pharmaceuticals. J.J.H. is supported by the Novo Nordisk Foundation Center for Basic Metabolic Research.

Received: November 8, 2021
Revised: January 24, 2022
Accepted: February 28, 2022
Published: March 30, 2022

REFERENCES

1. Müller, T.D., Nogueiras, R., Andermann, M.L., Andrews, Z.B., Anker, S.D., Argente, J., Batterham, R.L., Benoit, S.C., Bowers, C.Y., Broglio, F., et al. (2015). Ghrelin. Mol. Metab. *4*, 437–460.
2. Kojima, M., Hosoda, H., Date, Y., Nakazato, M., Matsuo, H., and Kangawa, K. (1999). Ghrelin is a growth-hormone-releasing acylated peptide from stomach. Nature *402*, 656–660.
3. Ariyasu, H., Takaya, K., Tagami, T., Ogawa, Y., Hosoda, K., Akamizu, T., Suda, M., Koh, T., Natsui, K., Toyooka, S., et al. (2001). Stomach is a major source of circulating ghrelin, and feeding state determines plasma ghrelin-like immunoreactivity levels in humans. J. Clin. Endocrinol. Metab. *86*, 4753–4758.
4. Mani, B.K., Puzziferri, N., He, Z., Rodriguez, J.A., Osborne-Lawrence, S., Metzger, N.P., Chhina, N., Gaylinn, B., Thorner, M.O., Thomas, E.L., et al. (2019). LEAP2 changes with body mass and food intake in humans and mice. J. Clin. Invest. *129*, 3909–3923.
5. Ge, X., Yang, H., Bednarek, M.A., Galon-Tilleman, H., Chen, P., Chen, M., Lichtman, J.S., Wang, Y., Dalmas, O., Yin, Y., et al. (2018). LEAP2 is an endogenous antagonist of the ghrelin receptor. Cell Metab. *27*, 461–469.e6.
6. M'Kadmi, C., Cabral, A., Barrile, F., Giribaldi, J., Cantel, S., Damian, M., Mary, S., Denoyelle, S., Dutertre, S., Péraldi-Roux, S., et al. (2019). N-terminal liver-expressed antimicrobial peptide 2 (LEAP2) region exhibits inverse agonist activity toward the ghrelin receptor. J. Med. Chem. *62*, 965–973.
7. Hagemann, C.A., Zhang, C., Hansen, H.H., Jorsal, T., Rigbolt, K.T.G., Madsen, M.R., Bergmann, N.C., Heimbürger, S.M.N., Falkenhahn, M., Theis, S., et al. (2021). Identification and metabolic profiling of a novel human gut-derived LEAP2 fragment. J. Clin. Endocrinol. Metab. *106*, e966–e981.
8. Baldini, G., and Phelan, K.D. (2019). The melanocortin pathway and control of appetite-progress and therapeutic implications. J. Endocrinol. *241*, R1–R33.
9. Maciejewski, M.L., Arterburn, D.E., Van Scoyoc, L., Smith, V.A., Yancy, W.S., Weidenbacher, H.J., Livingston, E.H., and Olsen, M.K. (2016). Bariatric surgery and long-term durability of weight loss. JAMA Surg. *151*, 1046.

10. Moffett, R.C., Docherty, N.G., and le Roux, C.W. (2021). The altered enteroendocrine reportoire following roux-en-Y-gastric bypass as an effector of weight loss and improved glycaemic control. Appetite *156*, 104807.

11. Svane, M.S., Jørgensen, N.B., Bojsen-Møller, K.N., Dirksen, C., Nielsen, S., Kristiansen, V.B., Toräng, S., Wewer Albrechtsen, N.J., Rehfeld, J.F., Hartmann, B., et al. (2016). Peptide YY and glucagon-like peptide-1 contribute to decreased food intake after Roux-en-Y gastric bypass surgery. Int. J. Obes. *40*, 1699–1706.

12. Cornejo, M.P., Mustafá, E.R., Cassano, D., Banères, J.L., Raingo, J., and Perello, M. (2021). The ups and downs of growth hormone secretagogue receptor signaling. FEBS J. *288*, 7213–7229.

13. Druce, M.R., Wren, A.M., Park, A.J., Milton, J.E., Patterson, M., Frost, G., Ghatei, M.A., Small, C., and Bloom, S.R. (2005). Ghrelin increases food intake in obese as well as lean subjects. Int. J. Obes. *29*, 1130–1136.

14. Islam, M.N., Mita, Y., Maruyama, K., Tanida, R., Zhang, W., Sakoda, H., and Nakazato, M. (2020). Liver-expressed antimicrobial peptide 2 antagonizes the effect of ghrelin in rodents. J. Endocrinol. *244*, 13–23.

15. Shankar, K., Metzger, N.P., Singh, O., Mani, B.K., Osborne-Lawrence, S., Varshney, S., Gupta, D., Ogden, S.B., Takemi, S., Richard, C.P., et al. (2021). LEAP2 deletion in mice enhances ghrelin's actions as an orexigen and growth hormone secretagogue. Mol. Metab. *53*, 101327.

16. Barrile, F., M'Kadmi, C., De Francesco, P.N., Cabral, A., García Romero, G., Mustafá, E.R., Cantel, S., Damian, M., Mary, S., Denoyelle, S., et al. (2019). Development of a novel fluorescent ligand of growth hormone secretagogue receptor based on the N-Terminal Leap2 region. Mol. Cell. Endocrinol. *498*, 110573.

17. Gupta, D., Dowsett, G.K.C., Mani, B.K., Shankar, K., Osborne-Lawrence, S., Metzger, N.P., Lam, B.Y.H., Yeo, G.S.H., and Zigman, J.M. (2021). High coexpression of the ghrelin and LEAP2 receptor GHSR with pancreatic polypeptide in mouse and human islets. Endocrinology *162*, bqab148.

18. Cornejo, M.P., Castrogiovanni, D., Schiöth, H.B., Reynaldo, M., Marie, J., Fehrentz, J.A., and Perello, M. (2019). Growth hormone secretagogue receptor signalling affects high-fat intake independently of plasma levels of ghrelin and LEAP2, in a 4-day binge eating model. J. Neuroendocrinol. *31*, e12785.

19. Levin, F., Edholm, T., Schmidt, P.T., Grybäck, P., Jacobsson, H., Degerblad, M., Höybye, C., Holst, J.J., Rehfeld, J.F., Hellström, P.M., et al. (2006). Ghrelin stimulates gastric emptying and hunger in normal-weight humans. J. Clin. Endocrinol. Metab. *91*, 3296–3302.

20. Willems, M., Otto Quartero, A., and Numans, M.E. (2001). How useful is paracetamol absorption as a marker of gastric emptying? A systematic literature study. Dig. Dis. Sci. *46*, 2256–2262.

21. Takaya, K., Ariyasu, H., Kanamoto, N., Iwakura, H., Yoshimoto, A., Harada, M., Mori, K., Komatsu, Y., Usui, T., Shimatsu, A., et al. (2000). Ghrelin strongly stimulates growth hormone (GH) release in humans. J. Clin. Endocrinol. Metab. *85*, 4908–4911.

22. Traynard, P., Ayral, G., Twarogowska, M., and Chauvin, J. (2020). Efficient pharmacokinetic modeling workflow with the MonolixSuite: a case study of remifentanil. CPT Pharmacometrics Syst. Pharmacol. *9*, 198–210.

23. Flint, A., Raben, A., Astrup, A., and Holst, J.J. (1998). Glucagon-like peptide 1 promotes satiety and suppresses energy intake in humans. J. Clin. Invest. *101*, 515–520.

24. Fujikawa, T. (2021). Central regulation of glucose metabolism in an insulin-dependent and -independent manner. J. Neuroendocrinol. *33*, e12941.

25. Møller, N., and Jørgensen, J.O.L. (2009). Effects of growth hormone on glucose, lipid, and protein metabolism in human subjects. Endocr. Rev. *30*, 152–177.

26. DiGruccio, M.R., Mawla, A.M., Donaldson, C.J., Noguchi, G.M., Vaughan, J., Cowing-Zitron, C., van der Meulen, T., and Huising, M.O. (2016). Comprehensive alpha, beta and delta cell transcriptomes reveal that ghrelin selectively activates delta cells and promotes somatostatin release from pancreatic islets. Mol. Metab. *5*, 449–458.

27. Adriaenssens, A.E., Svendsen, B., Lam, B.Y.H., Yeo, G.S.H., Holst, J.J., Reimann, F., and Gribble, F.M. (2016). Transcriptomic profiling of pancreatic alpha, beta and delta cell populations identifies delta cells as a principal target for ghrelin in mouse islets. Diabetologia *59*, 2156–2165.

28. Lawlor, N., George, J., Bolisetty, M., Kursawe, R., Sun, L., Sivakamasundari, V., Kycia, I., Robson, P., and Stitzel, M.L. (2017). Single-cell transcriptomes identify human islet cell signatures and reveal cell-type-specific expression changes in type 2 diabetes. Genome Res. *27*, 208–222.

29. Svendsen, B., and Holst, J.J. (2021). Paracrine regulation of somatostatin secretion by insulin and glucagon in mouse pancreatic islets. Diabetologia *64*, 142–151.

30. Lauritzen, E.S., Jørgensen, J.O.L., Møller, N., Nielsen, S., and Vestergaard, E.T. (2020). Increased lipolysis after infusion of acylated ghrelin: a randomized, double-blinded placebo-controlled trial in hypopituitary patients. Clin. Endocrinol. (Oxf). *93*, 672–677.

31. Nass, R., Farhy, L.S., Liu, J., Prudom, C.E., Johnson, M.L., Veldhuis, P., Pezzoli, S.S., Oliveri, M.C., Gaylinn, B.D., Geysen, H.M., et al. (2008). Evidence for acyl-ghrelin modulation of growth hormone release in the fed state. J. Clin. Endocrinol. Metab. *93*, 1988–1994.

32. Hartman, M.L., Iranmanesh, A., Thorner, M.O., and Veldhuis, J.D. (1993). Evaluation of pulsatile patterns of growth hormone release in humans: a brief review. Am. J. Hum. Biol. *5*, 603–614.

33. Zigman, J.M., Nakano, Y., Coppari, R., Balthasar, N., Marcus, J.N., Lee, C.E., Jones, J.E., Deysher, A.E., Waxman, A.R., White, R.D., et al. (2005). Mice lacking ghrelin receptors resist the development of diet-induced obesity. J. Clin. Invest. *115*, 3564–3572.

34. Flint, A., Raben, A., Blundell, J.E., and Astrup, A. (2000). Reproducibility, power and validity of visual analogue scales in assessment of appetite sensations in single test meal studies. Int. J. Obes. *24*, 38–48.

35. Reeves, M.M., Davies, P.S.W., Bauer, J., and Battistutta, D. (2004). Reducing the time period of steady state does not affect the accuracy of energy expenditure measurements by indirect calorimetry. J. Appl. Physiol. *97*, 130–134.

36. Sanchez-Delgado, G., Alcantara, J.M.A., Ortiz-Alvarez, L., Xu, H., Martinez-Tellez, B., Labayen, I., and Ruiz, J.R. (2018). Reliability of resting metabolic rate measurements in young adults: impact of methods for data analysis. Clin. Nutr. *37*, 1618–1624.

37. Fuller-Jackson, J.P., Dordevic, A.L., Clarke, I.J., and Henry, B.A. (2020). Effect of sex and sex steroids on brown adipose tissue heat production in humans. Eur. J. Endocrinol. *183*, 343–355.

38. El Hadi, H., Frascati, A., Granzotto, M., Silvestrin, V., Ferlini, E., Vettor, R., and Rossato, M. (2016). Infrared thermography for indirect assessment of activation of brown adipose tissue in lean and obese male subjects. Physiol. Meas. *37*, N118–N128.

39. Thuzar, M., Law, W.P., Ratnasingam, J., Jang, C., Dimeski, G., and Ho, K.K.Y. (2018). Glucocorticoids suppress brown adipose tissue function in humans: a double-blind placebo-controlled study. Diabetes Obes. Metab. *20*, 840–848.

40. Orskov, C., Jeppesen, J., Madsbad, S., and Holst, J.J. (1991). Proglucagon products in plasma of noninsulin-dependent diabetics and nondiabetic controls in the fasting state and after oral glucose and intravenous arginine. J. Clin. Invest. *87*, 415–423.

41. Gregersen, N.T., Flint, A., Bitz, C., Blundell, J.E., Raben, A., and Astrup, A. (2008). Reproducibility and power of ad libitum energy intake assessed by repeated single meals. Am. J. Clin. Nutr. *87*, 1277–1281.

STAR★METHODS

KEY RESOURCES TABLE

REAGENT or RESOURCE	SOURCE	IDENTIFIER
**Antibodies**		
LEAP2 antibody	Caslo, Technical University of Denmark, DTU-Science Park	N/A
**Chemicals, peptides, and recombinant proteins**		
LEAP2 peptide (human studies)	Vivitide (previously named Peptides International)	N/A
Saline (9 mg/mL)	Fresenius Kabi	N/A
Human albumin (5%)	CSL Behring	Cat# 22,203
LEAP2 peptide (mice studies)	Novo Nordisk	N/A
Nutridrink	Nutricia	Cat# 579,760
Pefabloc	Merck Millipore	Cat# 30,827-99-7
HCl	Merck Millipore	N/A
**Critical commercial assays**		
LEAP2 radioimmunoassay	Department of Biomedical Sciences, University of Copenhagen, Copenhagen, Denmark	Cat# LEAP2_SB41
Acylghrelin radioimmunoassay	Merck Millipore	Cat# GHRA-88HK
Mouse/Rat Insulin Kit	Meso Scale Discovery	Cat# K152BZC-1
Immunodiagnostic system-iSYS assay	IDS Limited	Cat# IS-3700
Glucagon C-terminal directed antiserum	Department of Biomedical Sciences, University of Copenhagen, Copenhagen, Denmark	Cat# 4305
**Experimental models: Organisms/strains**		
Wild C57/BL6 mice	Janvier labs	N/A
GHSR-null mice	J. Zigman	N/A
**Software and algorithms**		
Prism (v9.0.0 and v9.1.1)	GraphPad software	N/A
FLIR tools program	FLIR systems	N/A
Monolix (version 2019R1)	Lixoft SAS	N/A
**Other**		
Indirect calorimetry (Vyntus CPX Canopy)	Vyaire Medical	N/A
Thermal imaging camera	FLIR Systems	Cat# A655sc

RESOURCE AVAILABILITY

Lead contact
Further information and requests for resources and reagents should be directed to and will be fulfilled by the lead contact, Filip K. Knop (filip.krag.knop.01@regionh.dk).

Materials availability
This study did not generate new unique reagents.

Data and code availability
- All data reported in this paper will be shared by the lead contact upon request
- This paper does not report original code
- Any additional information required to reanalyze the data reported in this work paper is available from the Lead Contact upon request

EXPERIMENTAL MODEL AND SUBJECT DETAILS

Human participants

Twenty young men were included. Inclusion criteria were Caucasian male aged 18–25 years with a body mass index of 20–30 kg/m^2. Exclusion criteria included anemia, serum creatinine above the normal range and/or albuminuria, history of hepatobiliary and/or gastrointestinal disorder and/or plasma liver enzymes (alanine or aspartate aminotransferases) above two times the normal range, allergy or intolerance to ingredients included in the standardized meals, first-degree relatives with diabetes and/or hemoglobin A_{1c} >48 mmol/mol [6.5%], regular tobacco smoking and/or use of other nicotine-containing products and any physical or psychological condition or ongoing medication that the investigators evaluated would interfere with trial participation.

Ethics approval

The study was approved by the Scientific-Ethical Committee of the Capital Region of Denmark (registration number H-19038628) and the Danish Data Protection Agency (approval number P-2020-125). The study was conducted in accordance with the Declaration of Helsinki (7th revision, 2013) and registered at ClinicalTrials.gov (registration numbers NCT04621409 and NCT04897984). Informed consent was obtained from all participants before inclusion.

Mouse models

All animals had *ad libitum* access to water and a chow diet (Altromin, 1310, Brogaarden, Denmark) and were housed in a 22°C temperature-controlled environment under a 12:12 h light-dark cycle (lights on from 6 AM to 6 PM) unless otherwise stated. All animal studies were approved by the Danish Animal Experiments Inspectorate and performed according to institutional guidelines. Animal licenses: 2017-15-0202-00119 and 2019-15-0201-00289. Wild C57/BL6 mice were purchased from Janvier labs (Le Genest-Saint-Isle, France). GHSR-null mice were kindly donated by J. Zigman and have been used in a previous publication.[33] All mice had two weeks to habituate in their corresponding cages before start of studies.

METHOD DETAILS

Peptides and preparation of infusions

Human LEAP2 was synthesized by Vivitide (Louisville, Kentucky, USA). The peptide was confirmed to be identical to the natural peptide by high-performance liquid chromatography and mass spectrometry, amino acid sequencing and elemental analysis by the manufacturer. The final purity was >98%. The LEAP2 was dissolved in saline (9 mg/mL) with 0.5% human albumin, sterile filtrated and tested for endotoxins and sterility by the Capital Region Pharmacy (Herlev, Denmark) before dispensed into vials stored at −20°C until use. To prevent oxidation of methionine in the LEAP2 amino acid sequence, this was performed under a flow of nitrogen, and vials were blanked with nitrogen before filling. On experimental days, infusions were prepared by an unblinded staff member diluting LEAP2 to a total volume of 500 mL in saline (9 mg/mL, Fresenius Kabi, Uppsala, Sweden) containing 0.5% human albumin (5% solution, CSL Behring, Lyngby, Denmark). Placebo infusions consisted of 500 mL saline with 0.5% human albumin. Apart from preparing the infusion solutions, the unblinded staff member was not involved in the study. The infusion rate of LEAP2 in humans was estimated from a previous study of mice subjected to a single intravenous dose of LEAP2.[5] Synthetic LEAP2 used for animal studies was synthesized by Novo Nordisk (Bagsværd, Denmark), confirmed identical to the natural peptide by high-performance liquid chromatography and dissolved in phosphate buffer (50 mmol/L; pH 7.4) and saline (70 mmol/L).

Clinical study design and experimental procedures

The study was designed as a randomized, double-blind, placebo-controlled, crossover study and included two experimental days with intravenous infusion of LEAP2 (~25 pmol/kg/min = 115 ng/kg/min) or placebo (saline) with minimum one week between visits. A prespecified randomization list was generated from www.sealedenvelope.com with a block size of four. The study was conducted from October 2020 to February 2021 at Center for Clinical Metabolic Research, Copenhagen University Hospital — Herlev and Gentofte, Copenhagen, Denmark. See Figure 1 for an overview of the clinical study design. Each experimental day was preceded by 12 h of fasting including any liquids and 48 h of abstinence from alcohol, strenuous physical activity, medicine and intermittent fasting and/or excessive eating. Participants were instructed to keep a food diary for 48 h prior to experimental days. No experimental days were planned on Mondays since weekend activities often differ from activities during weekdays. In the evening before the experimental days (between 6 and 8 PM), the participants consumed a standardized meal (558 g pasta Bolognese; same energy content per 100 g as the *ad libitum* meal) and were asked to remain fasting until the experimental day. At the beginning of each experimental day, a cannula was inserted in a cubital vein in each arm, one for blood sampling and one for peptide infusion. The forearm for blood sampling was wrapped in a heating pad (45°C) to arterialize the venous blood. LEAP2 or placebo infusion was started at timepoint 0 min. At time 65–70 min, the participants were given a liquid mixed meal (2.97 mL/kg body weight; 1,010 kJ, 29.7 g carbohydrate, 9.6 g protein and 9.3 g fat per 100 mL; Nutricia Nutridrink, Allerød, Denmark) mixed with 1.5 g acetaminophen plus 50 mL of water. An *ad libitum* meal was served 260 min after start of the infusion and consisted of pasta Bolognese (565 kJ, 15.0 g carbohydrate, 5.3 g protein and 5.6 g fat per 100 mL) together with 400 mL of water. The *ad libitum* meal was served in undisturbed surroundings, and participants were instructed to eat until they felt comfortably full. The infusion was terminated when participants had finished eating.

Blood samples were collected at timepoints −35, −10, 0, 15, 30, 45, 60, 75, 90, 105, 135, 165, 195 and 255 min (for all analyses) as well as at timepoints 120, 150, 180 and 225 min (for analysis of plasma glucose only).

Visual analog scale ratings

Sensations of hunger, satiety, prospective food consumption, fullness, nausea, comfort and thirst were rated on 100 mm VAS at timepoints −35, 0, 30, 75, 105, 135, 165, 195, 225 and 255 min and after the *ad libitum* meal.[34] After eating, participants evaluated the *ad libitum* meal by VAS with a score (median (interquartile range)) for appearance of 55 (36–72) mm, taste 65 (42–76) mm, off-notes 17 (7–31) mm, smell 63 (50–72) mm and overall impression 59 (45–73) mm indicating a moderate evaluation of the meal.

Indirect calorimetry

Resting energy expenditure and respiratory quotient were measured by indirect calorimetry (Vyntus CPX Canopy; Vyaire Medical, Höchberg, Germany) for 15 min at timepoints −25 (baseline), 45, 120 and 240 min, with the participants awake and in a supine position from 5 min prior to each test. Participants rested in a hospital bed for 30 min before the first test and throughout the experimental day. The gas and flow analyzers were calibrated before each test. The first 5 min and the last minute of data collection were subsequently discarded, and the mean of a 5-min steady state period defined as a coefficient of variation <10% for VO_2 and CO_2 was used to estimate resting energy expenditure and respiratory quotient for each test. The 5-min steady state period with the lowest resting energy expenditure and with a respiratory quotient between 0.7 and 1.0 was considered. In a few cases without a 5-min steady state period, a 4-min steady state period was considered.[35,36] In the present study, a steady state period of 5 min was successfully reached in 135 out of 160 tests. One participant was excluded from the subsequent data analysis due to no steady state period in any of the four tests during an experimental day. A 4-min steady state period was considered in six cases reaching a total of 23 excluded tests from the data analysis.

Thermal imaging

To assess BAT thermogenesis, temperature of the upper chest was measured from −10 to 0 min, 0 to 15 min, 90 to 105 min and 180 to 195 min using a thermal imaging camera (FLIR A655sc; FLIR Systems, Wilsonville, Oregon, USA). Participants were positioned approximately one meter from the camera in a hospital bed with slight elevation of the head and wearing long-sleeved shirts that exposed the upper chest. The emissivity for measurement of human skin temperatures was set to 0.98 as detailed by the manufacturer. Thermal images were collected at 30-s intervals and analyzed using the FLIR tools program (FLIR Systems, Wilsonville, Oregon, USA). *Post hoc*, the two supraclavicular regions and a region corresponding to the manubrium of the sternum were marked as regions of interest and the maximum temperature per image was recorded. An average of records from the two supraclavicular regions was used. The manubrium region was used as a negative control reflecting the overall cutaneous heat production.[37–39] For analysis of BAT thermogenesis, manubrium-corrected temperature was calculated as the average of the supraclavicular skin temperatures minus the manubrium skin temperature. The first and last minutes of each measurement period were omitted from analysis. Average room temperature was 22.5 ± 0.06 °C adjusted by two air conditioners (Electrolux EXP35U538CW; AB Electrolux, Stockholm, Sweden) and recorded four times during each experimental day. Average humidity was 31.2 ± 0.39%. Due to technical issues with the camera, data from 3 of 40 experimental days are missing.

Urine sampling

To assess the amount of protein breakdown after the liquid mixed meal, urine samples were collected at timepoints 135 and 255 min for measurement of renal urea excretion and total urine production. At timepoint 60 min, 5 min prior to ingestion of the liquid mixed meal, participants were asked to empty their urinary bladder. Urine samples were stored at −80°C until analysis, and urine urea was measured by spectrophotometric methods following enzymatic hydrolysis and oxidation (Siemens Atellica CH 930; Siemens Healthcare, Ballerup, Denmark).

Human plasma analyses

For analysis of plasma glucose, blood was distributed in fluoride heparin-coated tubes, centrifuged immediately for 30 s (12,100 *g*, room temperature) and measured bedside by the glucose oxidase method (YSI 2900 Biochemistry Analyzer; YSI Incorporated, Yellow Springs, Ohio, USA). For analysis of LEAP2, blood was collected in chilled K_3EDTA tubes each containing 250 KIU of aprotinin. For analysis of acyl ghrelin, blood was collected in chilled K_2EDTA tubes added 20 μL pefabloc (100 mg/mL; Merck Millipore, Burlington, Massachusetts, USA). Subsequently, pefabloc-treated plasma was acidified with HCl (12.5 μL 2M HCl per 500 μL plasma; Merck Millipore, Burlington, Massachusetts, USA). For analysis of glucagon, blood was collected in K_2EDTA tubes. For analysis of GH, insulin, C-peptide, free fatty acids, glycerol, triglycerides and acetaminophen (paracetamol), blood was collected in tubes with lithium and heparin. All tubes were immediately cooled on ice, centrifuged for 15 min (2,000 *g*, 4°C) and stored at −80°C until analysis. LEAP2 concentrations were determined by an in-house radioimmunoassay directed against the N-terminal part of LEAP2. LEAP-2 was used for standards, and the tracer was a peptide composed of the 12 N-terminal amino acids of LEAP2, with a ^{125}I-labelled tyrosine added to the C-terminal end. The precision and accuracy of the assay were evaluated based on European Medicines Agency's guidelines for bioanalytical assay validation. The detection range of the assay was 5–500 pmol/L. Intra- and inter-assay variation was below 12%. Acyl ghrelin was measured by use of a radioimmunoassay kit (GHRA-88HK, Merck Millipore, Burlington, Massachusetts,

USA), which utilizes a specific antibody for the biologically active form of ghrelin with the octanoyl group on serine 3. Due to supply chain disruptions from the COVID-19 pandemic, acyl ghrelin was only measured in plasma samples at timepoints 0, 60, 105, 165 and 255 min. All quality controls fell within the prespecified quality control range. Plasma GH was measured by Immunodiagnostic system-iSYS assay (IS-3700; IDS Limited, Boldon, UK). The assay is based on chemiluminesence technology and was performed according to the manufacturer'|'s instructions. All quality controls (code number 40350; Bio-Rad, Copenhagen, Denmark) fell within the calculated quality control range. For analysis of glucagon, plasma was extracted in a final concentration of 70% ethanol. Glucagon was measured using a C-terminal directed antiserum (code number 4305) measuring glucagon of pancreatic origin as previously described.[40] Sensitivity for the assay was below 1 pmol/L, and intra-assay coefficient of variation was below 10%. Plasma insulin and C-peptide were measured using a two-sided electrochemiluminescence immunoassay (Atellica IM Analyzer; Siemens Healthcare, Ballerup, Denmark). Plasma triglycerides and acetaminophen were analyzed by spectrophotometric methods following enzymatic hydrolysis and oxidation (Siemens Atellica CH 930; Siemens Healthcare, Ballerup, Denmark). Plasma concentrations of free fatty acids and glycerol were determined by enzymatic methods modified to run on a COBAS 6000 automatic analyzer (Roche, Rødovre, Denmark) using a NEFA-HR assay (Wako Chemicals, Neuss, Germany) and glycerol assay (Randox Laboratories, Crumlin, UK), respectively.

Pharmacokinetic parameters of LEAP2 in healthy men

Eight healthy men, recruited among participants in the placebo-controlled, crossover study, were included in an additional clinical study investigating the kinetic parameters of exogenous LEAP2. Each participant underwent a single experimental day with intravenous infusion of LEAP2 (10 min at a rate of ~101 pmol/kg/min followed by 110 min at ~25 pmol/kg/min) (see Figure S1B for an overview of the clinical study design). The study was conducted from May 2021 to June 2021 at Center for Clinical Metabolic Research, Copenhagen University Hospital — Herlev and Gentofte, Copenhagen, Denmark. Fasting schedule for participants and all instructions from 48 h prior to the experimental day were similar to the placebo-controlled, crossover study. On the morning of the experimental day, a cannula was inserted in a cubital vein in each arm of the participant, one for blood sampling and one for peptide infusion. Throughout the experimental day, the forearm for blood sampling was wrapped in a heating pad (45°C) for collection of arterialized blood samples. LEAP2 infusion was started at timepoint 0 min and discontinued after 120 min. Blood samples were drawn every 15 min from timepoint −30 to 270 min and, additionally, at timepoints 118, 122, 124, 127 and 130 min. Plasma glucose was measured bedside every 15 min from timepoint −30 to 180 min and every 30 min from timepoint 180 to 270 min with a mean concentration of 4.85 ± 0.02 mmol/L. For analytical procedures related to plasma glucose and LEAP2 concentrations, please see the section "Human plasma analyses". Every 30 min from time −30 to 270 min, participants rated appetite-related sensations on 100 mm VAS[34] with a score (median (interquartile range)) for hunger of 72 (59–79) mm, prospective food consumption 73 (65–82) mm, satiety 22 (14–33) mm, fullness 21 (12–31) mm, comfort 78 (72–90) mm, thirst 78 (71–89) mm and nausea 3.0 (0–9.0).

Effects of LEAP2 on glucose metabolism and food intake in mice

Food intake and blood glucose measurements were measured in female GHSR-null mice (n = 8) and wild-type littermates (n = 10) (14–24 weeks of age). Mice were double-housed with matching genotype and age. All mice were fasted for four hours and randomized by mean body weight per cage and allocated into either vehicle (saline) or LEAP2 administration on the first day of the study. LEAP2 or vehicle was administered subcutaneously just before start of dark phase (6 PM). By start of dark phase, mice had *ad libitum* access to water and a normal chow diet. Cumulative food intake was measured one, two and four hours after administration and calculated as the average food intake per cage per g body weight of each mouse, respectively. Blood glucose was measured in blood from a tail puncture at baseline and one, two and four hours after administration by use of a glucometer (Contour ™xt meter; Ascensia Diabetes Care, Basel, Switzerland). One week later, mice underwent the same procedure, but with the opposite treatment. The concentration of the LEAP2 dose was 3 μmol/kg.

Effects of LEAP2 on insulin and plasma glucose concentrations in fasted mice

For fasting insulin and glucose concentrations, females (15 weeks of age) were used. Mice were housed four mice per cage. On the experimental day, mice were fasted from four hours before subcutaneous administration until end of the study and randomized by body weight into either vehicle (saline) or LEAP2 dosing (3 μmol/kg). Blood samples were collected at timepoints −60, 20 and 60 min from the retro-orbital vein plexus using EDTA-coated capillary tubes. Plasma was subsequently separated from the blood after centrifugation (3,000 *g*, 4°C, 5 min) and stored at −80°C until analysis. Blood glucose was measured at timepoints −60, 20 and 60 min from eye blood and at timepoint 0 min from a tail puncture by use of the same glucometer (Contour ™xt meter; Ascensia Diabetes Care, Basel, Switzerland). Mouse plasma insulin concentrations were determined by use of a Mouse/Rat Insulin Kit (cat: K152BZC-1; Meso Scale Discovery, Gaithersburg, Maryland, USA). The assay was performed according to the manufacturer's instructions, and the plate was measured by use of MESO QuickPlex SQ 120.

QUANTIFICATION AND STATISTICAL ANALYSIS

Unless otherwise stated, results are presented as mean ± SEM. Baseline values were calculated as a mean of timepoints −35, −10 and 0 min or, when only available −10 and 0 min. Baseline values for the postprandial period were calculated as a mean of timepoint

60 min. Calculations of AUC were based on the trapezoidal rule. AUC values are reported for the period 0 to 255 min. To assess the fasting and postprandial periods separately, AUC values are reported for the periods 0 to 60 min and 60 to 255 min, respectively. Student's paired *t* test was used to test for differences in baseline, peak, time to peak, AUC and mean values and *ad libitum* food intake in men. Indirect calorimetry and thermal imaging data were fitted to a repeated-measures mixed effects model with the intervention and timepoints as independent variables with interaction and Geisser-Greenhouse correction. Šidák's *post hoc* test was used to compare LEAP2 to placebo for each timepoint. For analysis of endogenous LEAP2 concentrations in response to a liquid mixed meal test, postprandial data during placebo infusion were fitted to a repeated-measures mixed effects model with time as a fixed effect and the individual participant as a random effect with Geisser-Greenhouse correction. Dunnett's post hoc test was used to compare endogenous LEAP2 concentrations at each timepoint (75 to 255 min) to the postprandial baseline. Mouse plasma glucose, food intake and insulin data were analyzed by a repeated-measures two-way ANOVA with Šidák's post hoc test. Statistical analyses were carried out using GraphPad Prism v9.0.0 and v9.1.1 (GraphPad Software, San Diego, California). A two-sided p value < 0.05 was considered statistically significant. The population size calculation was based on data from a previous study investigating the reproducibility of an *ad libitum* meal in healthy men.[41] Eighteen participants were needed to detect a difference of minimum 500 kJ in *ad libitum* food intake with a with-in subject difference of 739 kJ, a power of 80% and the above-mentioned significance level. To ensure sufficient power, 20 participants were included.

We constructed and applied a pharmacokinetic model to the data using the software Monolix (Monolix version 2019R1; Lixoft SAS, Antony, France). The parameters were estimated using the Stochastic Approximation EM algorithm.[22] The one-compartment model assumed input from both endogenous LEAP2 secretion and the exogenous LEAP2 infusion assuming that LEAP2 kinetics followed a single-exponential elimination. We assumed that the endogenous production of LEAP2 was constant i.e. not suppressed during infusion of exogenous LEAP2. This model probably represents a simplification of the actual physiological LEAP2 kinetics, but the data fitted well to model predictions.

Cell Reports Medicine, Volume *3*

Supplemental information

LEAP2 reduces postprandial glucose excursions and *ad libitum* food intake in healthy men

Christoffer A. Hagemann, Malene S. Jensen, Stephanie Holm, Lærke S. Gasbjerg, Sarah Byberg, Kirsa Skov-Jeppesen, Bolette Hartmann, Jens J. Holst, Flemming Dela, Tina Vilsbøll, Mikkel B. Christensen, Birgitte Holst, and Filip K. Knop

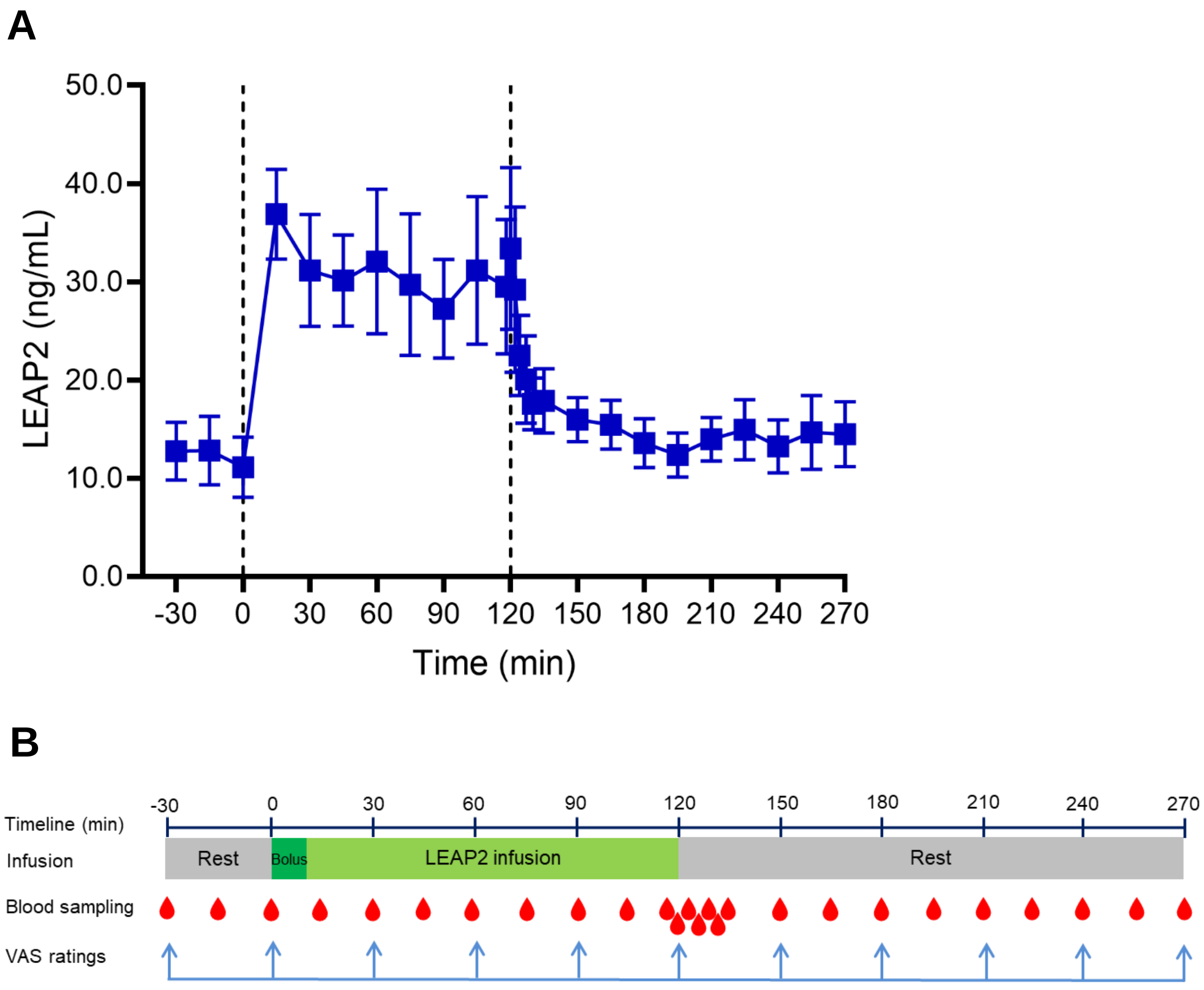

Figure S1. Clinical study describing pharmacokinetic parameters of exogenous LEAP2, Related to STAR Methods.

Plasma concentrations of LEAP2 (**A**) and overview of the clinical study design including eight healthy, young men (**B**). Bold dotted line, infusion start (0 min) and infusion stop (120 min). Data are presented as mean ± SEM. Abbreviations: LEAP2, liver-expressed antimicrobial peptide 2; VAS, visual analogue scale.
